# Machine Health Indicators and Digital Twins

**DOI:** 10.3390/s25072246

**Published:** 2025-04-02

**Authors:** Tal Bublil, Roee Cohen, Ron S. Kenett, Jacob Bortman

**Affiliations:** 1 BGU-PHM Laboratory, Department of Mechanical Engineering, Ben-Gurion University of the Negev, P.O. Box 653, Beer Sheva 8410501, Israel; talbub@post.bgu.ac.il (T.B.); jacbort@bgu.ac.il (J.B.); 2 KPA Ltd., and The Samuel Neaman Institute, Technion, Haifa 3200003, Israel; ron@kpa-group.com

**Keywords:** health indicators (HIs), digital twins (DTs), sensors, AI, condition-based maintenance (CBM), prognostics and health management, structural health monitoring

## Abstract

**Highlights:**

- Integration of health indicators (HIs) and digital twins (DTs) for predictive maintenance.
- Reliability of uncertainty quantification boost in health monitoring systems.
- Enhancement of hybrid model diagnostics, combining physics-based and data-driven approaches.

**Abstract:**

Health indicators (HIs) are quantitative indices that assess the condition of engineering systems by linking sensor data with monitoring, diagnostic, and prognostic methods to estimate the remaining useful life (RUL). Digital twins (DTs), which serve as digital representations of physical assets, enhance system monitoring, diagnostics, and prognostics by operationalizing analytic capabilities derived from sensor data. This paper explores the integration of HIs and DTs, illustrating their roles in condition-based maintenance and structural health monitoring. The methodologies discussed span data-driven and physics-based approaches, emphasizing their applications in rotary machinery, including bearings and gears. These approaches not only detect anomalies but also predict system failures through advanced modeling and machine learning (ML) techniques. The paper provides examples of HIs derived from vibration analysis and soft sensors and maps future research directions for improving health monitoring systems through hybrid modeling and uncertainty quantification. It concludes by addressing the challenges of data labeling and uncertainties and the role of HIs in advancing performance engineering, making DTs a pivotal tool in predictive maintenance strategies.

## 1. Introduction

The increasing complexity of modern engineering systems has stimulated the development of advanced condition-based maintenance (CBM) strategies to enhance reliability and efficiency. Traditional time-based maintenance approaches often result in unnecessary servicing or unexpected failures, leading to increased operational costs and downtime. To address these challenges, digital twins (DTs) have emerged as a transformative technology in Industry 4.0, enabling real-time system monitoring, diagnostics, and prognostics through the integration of health indicators (HIs) derived from sensor data and computational models.

DTs provide a virtual representation of physical assets that are continuously updated with live data to simulate, analyze, and optimize system performance. By leveraging statistical models, artificial intelligence (AI), and physics-based simulations, DTs facilitate predictive maintenance by identifying faults, estimating remaining useful life (RUL), and hence reduce downtime [1]. The integration of HIs—quantitative metrics derived from vibration analysis, thermal measurements, and other sensor inputs—further enhances the prognostic capabilities of DTs [2]. These indicators serve as key inputs for machine learning (ML) algorithms and statistical methods, which, in turn, generate actionable insights for maintenance decision-making [3].

Recent advancements in fault diagnosis and predictive maintenance have significantly benefited from the integration of entropy-based analysis and DT technologies. Jaen-Cuellar et al. [4] demonstrated that entropy-based HIs can enhance fault detection in induction motors by quantifying signal disorder. Additionally, Lv et al. [5] introduced a high-order synchroextracting transform to enhance time-frequency analysis for fault diagnosis in rotating machinery. Their method improves the characterization of amplitude-frequency modulation features, which are critical for identifying complex machine faults.

However, despite significant advancements, many challenges still remain, including precise classification of faults, accurate severity assessment, and efficient optimization of maintenance strategies. For example, in rotating machinery, distinguishing between different types of gear faults—such as pitting and chipped teeth—remains a key challenge due to their similar vibration signal characteristics [6]. Hybrid modeling approaches combining physics-based and data-driven methods to enhance interpretability and accuracy in predictive maintenance frameworks are a promising emerging research direction.

This paper explores the integration of HIs and DTs for predictive maintenance, focusing on their application in rotary machinery, including bearings and gears. We present a comprehensive review of data-driven and physics-based methodologies, discuss challenges related to data labeling and uncertainty quantification, and propose future research directions for advancing DT-enabled health monitoring systems. By addressing these challenges, DTs can redefine performance engineering, enabling more efficient and cost-effective condition-based maintenance strategies in complex engineering environments.

## 2. Health Indicators and Digital Twins

DTs are transforming condition-based maintenance in the heavy machinery industry by bridging the gap between real-world operations and advanced predictive analytics. This paradigm enables continuous monitoring, diagnosis, and performance prediction by integrating HIs with dynamic models, creating a digital counterpart of physical assets [7,8,9]. HIs are used in DTs to operationalize analytic capabilities derived from sensor data. For example, DTs have significantly enhanced maintenance capabilities in train systems by enabling real-time fault diagnosis and predictive maintenance, particularly through the integration of HIs. In the case of freight car braking systems, Davidyan et al. [10] developed an operational DT that employs pressure sensors and brake force measurements to monitor the braking system performance. The DT utilizes HIs such as brake cylinder pressure deviations, actuation response time, and pressure decay rates to detect leakage, delayed activation, and insufficient braking force. By continuously analyzing these indicators, the system can identify faults at an early stage, predict failures, and optimize maintenance scheduling, reducing the risk of unexpected breakdowns and improving overall train safety. Similarly, in locomotive parking brakes, DTs use sensor-based HIs to monitor brake engagement response, holding force consistency, and actuation patterns [11]. These indicators improve fault detection accuracy and facilitate data-driven predictive maintenance strategies, reducing unplanned interventions and enhancing railway operational efficiency.

One major conceptual movement outlined in this paper is the transition from the engineering of design to the engineering of performance [1]. DTs and HIs play a central role in this transition. HIs can be directly measured or derived from sensor inputs, like in soft sensors. Moreover, one can invoke different models in monitoring, diagnostic, or prognostic applications. Monitoring can be conducted with statistical process monitoring methods, while diagnostics can be derived from the analysis of designed experiments of observed data. Prognostics are based on predictive analytics and reliability models. Prognostic and health management (PHM) requires timely predictions of RUL using data from sensors. To be able to predict RUL, a suitable HIs and a prognostic model are needed [12].

The development of predictive models for relevant end-product quality properties from data contained in large process histories, databases, or data lakes is an area where the number of deep AI/ML applications found in the literature is increasing. Quality properties tend to be available less frequently due to the nature of the associated measurement systems. These are usually obtained offline, often with significant delays and involving complex, expensive, and time-consuming experimental protocols. Given their importance, such variables are often the target for process supervision, monitoring, control, and optimization, where more frequent estimates are required. Soft sensors are inferential models developed to achieve this goal and bring other associated benefits, such as a reduction in the inspection overhead and improvements in the consistency of the final product quality and in process efficiency, among others. Soft sensors, like HIs, are based on process data; they are also sometimes referred to as process soft sensors. Many soft sensor models have been developed for both continuous and batch processes. Applications include the prediction of compositions from the outgoing streams in distillation columns, the prediction of the Research Octane Number (RON) in industrial catalytic reforming units, the estimation of cement properties, and the prediction of NO_x_ and CO_2_ emissions in industrial boilers and commercial ships [13,14,15].

HIs, soft sensors, and DTs are also applied in static systems such as bridges and buildings [16]. For an application of degradation models to estimate RUL, see [17].

## 3. Health Indicators in Rotary Machinery

Vibration analysis is widely used for predictive maintenance of rotating machinery [18,19,20,21]. It is most critical to monitor bearings and gears, the most common mechanical components in these machines. However, accurately measuring and quantifying the health of bearings and gears is challenging. To address this, numerous vibration-based methods have been developed to create HIs for these components. An HI for mechanical systems is a metric or parameter that provides real-time data on the condition and performance of a system or component, which can help to assess its current state and to predict potential failures. There are two primary approaches to achieving these goals: physics-based and data-driven [18,22,23,24,25,26,27].

The physics-based approach focuses on analyzing the dynamic behavior of a component under various conditions using theoretical models, such as dynamic models or finite element models [28,29,30,31,32]. These models are grounded in the principles of mechanics, dynamics, and material science, enabling a deep understanding of underlying fault mechanisms and different operating conditions. The key advantage of this approach is its interpretability, as operators can understand the physical causes of damage based on the model’s output. However, the models rely on broad assumptions in order to balance simplicity and realism, making them less applicable to complex systems.

**Figure 1:**
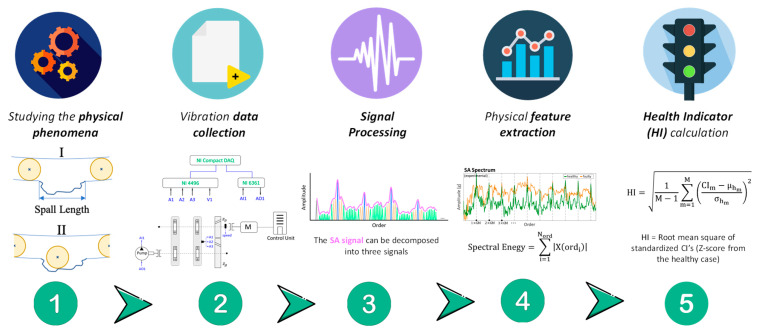
Flow diagram of the physics-based approach stages: (1) Study physical phenomena using dynamic models to analyze system response in healthy and faulty conditions; (2) Collect vibration data from the inspected machinery; (3) Apply signal processing techniques. (4) Extract meaningful physical features that emphasize faults; (5) Compute robust health indicators by combining extracted features.

**Figure 2:**
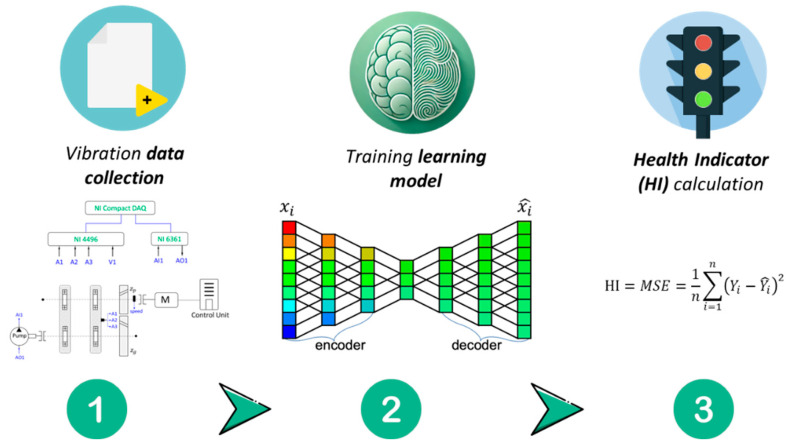
Flow diagram of the data-driven approach stages: (1) Collect vibration data from the inspected machinery; (2) Train an AI model to recognize patterns associated with healthy and faulty conditions; (3) Use the trained AI model to predict the condition of new samples.

Traditionally, physics-based approaches utilize signal processing techniques designed to highlight the physical behavior of faults in vibration signals and to extract meaningful features that can be combined into HIs for monitoring the health status of components. Figure 1 presents the common stages in the physics-based approaches. These indicators are typically well-defined physical quantities, such as vibration amplitude, frequency components, or energy levels, that correspond to specific mechanical phenomena. For example, in gears, the most common algorithm used is synchronous averaging (SA), which leverages the gear’s synchronous behavior to filter out noise and other components from the signal. From the SA signal and its variations, such as residual and difference signals, various condition indicators (like root mean square (RMS) or kurtosis) can be extracted to detect faults such as tooth breakage or pitting [22]. In bearings, the most common technique is envelope analysis, which amplifies the impact from bearing components and enables monitoring the relevant bearing tones [33,34,35]. On the other hand, the data-driven approach leverages statistical analysis, ML, and AI to diagnose faults by identifying patterns in large datasets of vibration signals without relying on specific system assumptions, as illustrated in Figure 2. It typically involves learning a complex function that transforms raw data or extracted features into a scalar HI, often using supervised or unsupervised learning models. This method is particularly effective for complex systems where deriving an accurate physical model is challenging. By analyzing historical data, data-driven approaches can detect anomalies and predict failures with high accuracy, as seen in studies using deep learning architectures to detect faults in rolling element bearings [36,37]. However, a significant drawback is the need for extensive labeled historical data across various conditions and fault modes. It is important to note that machines in heavy industries are typically healthy for most of their operational time, with faults occurring infrequently. Additionally, the HIs produced by data-driven methods are often holistic, capturing subtle changes in system behavior that may not immediately indicate a specific physical cause. While these HIs are widely used for detection in condition-based maintenance, challenges arise in more advanced stages like diagnostics and prognostics. The “black box” nature of data-driven methods limits transparency, making it difficult to directly correlate results with the machine’s physical state. This can hamper severity estimation across different machines, as the same HI may not consistently reflect the same underlying physics, limiting its usefulness in deeper maintenance analyses. However, the integration of these two approaches is increasingly seen to be a promising avenue for enhancing fault diagnosis in rotary machinery, combining the deep physical insights of physics-based models with the adaptive and predictive capabilities of data-driven techniques [38,39]. In this section, we discuss the importance of datasets and data labeling, along with the concept of HIs, focusing on their application in two distinct cases: bearing fault detection and gear defect analysis. Both are critical for mechanical systems and are supported by numerous examples.

### 3.1. Experimental Dataset Types and Data Labeling

When developing HIs, a reliable database is essential, regardless of the approach used. Typically, this database is created through controlled experiments, with the two main types being endurance tests and seeded tests. In seeded tests, faults are intentionally introduced into the test rig, and the system’s vibrations are measured under various conditions, such as changing speed or load [40,41]. This approach enables precise labeling of the data since the specific fault introduced is known for each measurement. However, introducing a new fault requires disassembling and reassembling the test rig, which can significantly alter the vibration signals and make it challenging to extract consistent features from the data. On the other hand, endurance tests involve introducing a small defect into a bearing or gear and running the system to failure [40,42,43]. In these experiments, the test rig typically remains assembled throughout the process, meaning that changes in the measurements are primarily due to the defect’s progression. While this method ensures that the observed changes are related to the evolving fault, it is difficult to estimate the exact condition of the fault during the intermediate stages of the experiment; only the initial and final states are known.

Labeling data in endurance tests is essential for both physics-based and data-driven approaches when developing HIs. Without proper labeling, it becomes challenging to accurately determine the severity of faults at various stages of the experiment, while a comprehensive HI should be able to assess fault severity well. However, without accurate labeling, it is difficult to link changes in the measured data to fault severity, especially in data-driven approaches where model accuracy heavily depends on the quality and specificity of labeled data. By assigning labels that correspond to different levels of fault severity throughout the test, models can learn to identify subtle changes in the system’s behavior. For physics-based approaches, labeled data are crucial for fine-tuning simulations and validating physical models against real-world conditions, ensuring that HIs accurately reflect the system’s physical state. Without proper labeling, both approaches risk producing less reliable indicators, limiting their effectiveness in early fault detection and accurate fault severity estimation. Additionally, neither approach can validate the results of their HIs without labeled data, making them unsuitable for real-life applications.

### 3.2. Example of Health Indicators in Bearings

HIs play a vital role in monitoring and diagnosing faults in rolling element bearings (REBs). To validate the effectiveness of the proposed health indicators (HIs), we present examples from both data-driven and physics-based approaches applied to bearing fault detection. These verification examples demonstrate how HIs can be constructed and used to detect faults and estimate severity in rolling element bearings (REBs).

One example of data-driven is the use of an Auto-Encoder (AE) architecture. AE is a specialized neural network architecture used for dimensionality reduction and fault detection in REBs [44,45,46,47]. The AE consists of an encoder that compresses the input data into a lower-dimensional representation and a decoder that reconstructs the data. The key concept behind this method is training the AE using data from a healthy bearing state. Once trained, the AE will yield a low reconstruction error (RE) when processing healthy signals, while signals from faulty bearings will result in a larger RE due to differences in distribution. The reconstruction error serves as an effective HI, with a higher error indicating a potential fault. This approach provides an anomaly score that quantifies the deviation from the healthy state, making it a reliable indicator for bearing fault detection.

**Figure 3:**
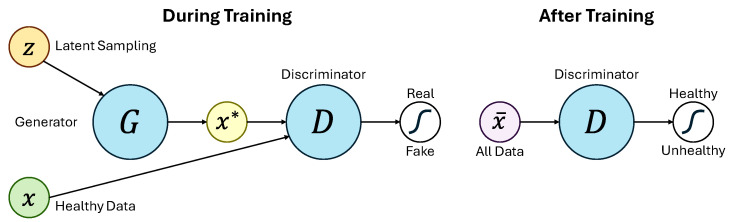
GAN process for training and detection based on [8].

Another validation example is based on Generative Adversarial Networks (GANs). GANs have emerged as another data-driven method for bearing fault diagnosis, as demonstrated in Figure 3 [8]. The architecture consists of two neural networks: a Generator G that produces signals similar to real data $x^{*}$, and a Discriminator D that distinguishes between real and generated signals. During training, the Discriminator learns to differentiate between healthy signals and those generated by the Generator. After training with healthy data, the Discriminator can be used to detect faults by comparing new signals to the healthy distribution. The HI derived from this process is calculated as HI=1−D(X), where D(X) represents the Discriminator’s confidence that a signal belongs to the healthy class. As the signal deviates from the healthy state, this HI increases, offering a robust indicator of potential faults.

**Figure 4:**
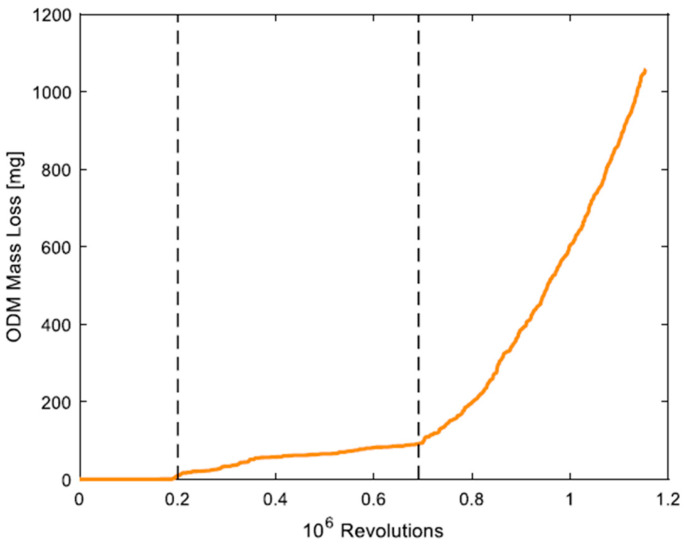
Mass loss versus revolutions, as presented in one of the experiments in [43]. The first vertical line represents the transition between the initiation and propagation phases, while the second vertical line estimates the transition from the steady-state to the accelerated stage.

A physics-based verification example uses Oil Debris Monitoring (ODM) to estimate spall size progression [43]. In this method, spall propagation is divided into three stages (presented in Figure 4), and an HI is provided for the accelerated damage growth stage. The model assumes that the spall’s cross-sectional area remains constant while accounting for bearing geometry, Hertzian contact mechanics, and spall geometry. The ODM sensor detects mass loss, which is then correlated with spall length, allowing for the estimation of bearing damage severity. This HI has been experimentally validated, showing accuracy within 13% of the actual spall angle. However, further research is needed to generalize this model, particularly during the steady-state propagation stage, in order to enhance its robustness.

One of the most common condition indicators in bearings is the set of four bearing tones, each corresponding to one of the main bearing components [33,35,48,49]. These frequencies can be calculated analytically, and they are extracted from the signal spectrum after physical signal processing that highlights the bearing’s contribution. Changes in the energy of these tones can indicate faults in specific components. Tracking these changes serves as an HI for both fault detection and classification [25,50]. However, due to bearing slippage, which causes components to become unsynchronized, the actual bearing tone frequencies may differ from their predicted values. Slippage, being chaotic, complicates the extraction of tone energy. To address this, the Bearing Tone Location (BTL) algorithm automatically searches for spectral patterns associated with the bearing tones [51]. This algorithm identifies the true location of the bearing tones and provides a confidence level for detection. By reducing the need for expert input, the BTL algorithm facilitates the automatic extraction of bearing tone energy, making it a valuable tool for fault diagnosis.

Despite significant advancements in bearing technology, a gap remains with regard to accurately estimating fault severity. To date, methods for severity estimation have been tailored to specific fault types, thereby limiting the robustness of the HI. Moreover, most research has focused on individual fault types without considering the dynamic interplay between multiple defects. In practice, the onset of one fault can trigger a chain reaction, leading to the development of additional faults. Consequently, the current HI may not adequately capture the true condition of the system in such scenarios.

### 3.3. Example of Health Indicators in Gears

This section provides verification examples demonstrating the effectiveness of health indicators (HIs) for diagnosing gear faults, using both experimental and hybrid model approaches.

A detailed verification example is provided in the work by Bachar et al. [52], which proposes a physics-based HI for diagnosing local tooth faults in spur and helical gears. The HI combines key features such as the RMS and kurtosis of the difference signal, as well as the RMS and skewness of its envelope, which reflect energy and peak sharpness—both typical indicators of gear faults. To create a single HI, the z-score distance of each feature from the healthy baseline was calculated and combined using a Euclidean norm. The proposed HI, tested on experimental datasets, successfully detects faults under varying speeds, loads, and fault severities. Bachar et al. [38] also proposed a hybrid physical AI-based strategy for fault detection and severity estimation. The fault-detection process relies on an unsupervised algorithm that uses only healthy measured data, as labeled faulty data are typically unavailable. They introduced an advanced technique to extract “sensitive” features from selective difference signals around each gear mesh harmonic, which are combined into the HI for improved early detection accuracy. For severity estimation, a supervised algorithm trained on healthy measured data and both healthy and simulated data follows a zero-shot learning approach, using another HI that aggregates features like RMS, spectral energy, kurtosis, and skewness. While less sensitive, this HI is used after a fault has already been detected, as the sensitive features may emphasize discrepancies between measured and simulated signals. Bansal et al. [53] applied Support Vector Machine (SVM) techniques to classify gear faults using vibration data. They assess SVM performance in identifying healthy gears and those with defects like chipped, missing, or worn teeth. The study introduces interpolation and extrapolation methods to address situations where training data is unavailable at certain speeds. In this approach, the SVM score represents the HI, and results show that SVMs, particularly with radial basis function kernels, accurately classify gear faults across varying speeds, enhancing the reliability of machine condition monitoring. Feng et al. [54] proposed a vibration-based prognostic scheme for monitoring and predicting gear surface wear progression in intelligent manufacturing systems. The study introduces a new HI that tracks gear wear severity by combining cyclic correntropy and Wasserstein distance, enabling real-time monitoring of wear-related signal changes. This HI is integrated into an optimized Gated Recurrent Unit network to predict the RUL of the gear system. The Gated Recurrent Unit network is optimized using a genetic algorithm to enhance the accuracy of the predictions. Matania et al. [55] proposed a hybrid, physical, AI-based approach to estimate the fault severity of tooth face faults in gears. They address the challenge of differences between simulated and experimental signals, which commonly hamper the application of machine learning in fault diagnosis. By leveraging both simulated and limited real-world data, the proposed method adapts physical models through domain adaptation, reducing the gap between simulated and experimental conditions. This is achieved by estimating and compensating for the transfer function effects between machines. The methodology includes signal preprocessing, followed by fault size estimation using a k-nearest neighbor’s model. The method effectively reduces prediction error even with a small amount of experimental faulty data, making it a robust solution for gear health management.

Despite these advancements, challenges remain in accurately classifying different types of local gear faults due to their similar vibration signal characteristics. For instance, while HIs exist to detect faults such as pitting and chipped teeth, distinguishing between them remains difficult. This classification challenge also impacts severity estimation, as most severity assessment algorithms are designed for specific fault types. Without precise fault classification, selecting the appropriate severity estimation model becomes problematic. Moreover, even for well-identified faults, severity estimation itself remains a challenge due to variations in fault progression and system dynamics. Addressing these issues requires further research in feature engineering, hybrid modeling, and advanced AI-driven classification techniques.

## 4. The Importance of Uncertainties

Incorporating and analyzing uncertainties in engineering models, simulations, HIs, and DTs is crucial for improving the reliability of predictions, especially in health monitoring and predictive maintenance. Uncertainties arise from modeling assumptions, measurement errors, operational variability, and unknown dynamics. Addressing these uncertainties may lead to enhancing the accuracy of models, particularly when using them for decisions related to HIs or DTs. This review examines various strategies for managing uncertainties, drawing insights from relevant literature.

Uncertainty quantification (UQ) is essential for evaluating engineering model reliability. In the field of fluid dynamics, the authors in [56] utilized large-eddy simulations with wall modeling to manage uncertainties associated with inadequate grid resolution, which is critical for capturing turbulent boundary behavior. This method balances computational cost and accuracy in high Reynolds number flows, addressing the limitations in simulations due to constrained resources.

Dynamic degradation modeling offers another approach for managing uncertainties, especially for systems with gradual performance deterioration. The authors in [17] introduced a nonparametric method based on functional variance processes to predict the RUL. Their approach models unit-specific degradation paths using functional principal component analysis, capturing variability and randomness in degradation, and thereby addressing uncertainties from diverse operational conditions. This method is valuable for predictive maintenance aimed at forecasting systems or component failures.

Data-driven models, fueled by real-time sensor data, have become popular for UQ due to their flexibility. The authors in [16] presented a semi-supervised deep learning framework for creating intelligent HI using structural health monitoring data. The framework incorporates prognostic criteria like monotonicity and trendability while leveraging both labeled and unlabeled data to handle uncertainties in incomplete datasets. This approach is effective for managing complex structures such as composites that often exhibit non-linear and unpredictable damage patterns. ML techniques, such as deep learning, allow for handling uncertainties related to data scarcity, varying damage scenarios, and sensor noise.

DTs face challenges in managing uncertainties due to modeling inaccuracies and parameter variability. The book Computational Modeling by Case Study: All Models Are Uncertain [57] details methodologies such as Bayesian methods, Monte Carlo simulations, and stochastic modeling employed to address these challenges. These probabilistic techniques help to continuously update DT models based on new data, refining predictions and maintaining model accuracy over time. Bayesian approaches, in particular, are beneficial for updating parameters and improving uncertainty estimates as new information is collected, thus addressing both aleatory (random) and epistemic (knowledge-related) uncertainties.

Addressing uncertainties in engineering models, HIs, and DTs requires integrating data-driven methods, hybrid modeling, and stochastic processes. Advances in ML, nonparametric approaches, and probabilistic techniques enhance the accuracy and robustness of predictive maintenance strategies. The incorporation of techniques that account for variability, randomness, and incomplete data increases the reliability of DTs and HIs. As engineering systems grow in complexity, the development of sophisticated UQ methods will be essential for optimizing maintenance strategies and decision-making processes.

## 5. Discussion

The interplay between HIs and DTs highlights a paradigm shift from traditional maintenance to predictive analytics-driven performance engineering. HIs provide real-time insights into system conditions, enabling accurate diagnostics and prognostics. In rotary machinery, for example, vibration-based methods for bearings and gears showcase the efficacy of physics-based and data-driven approaches in identifying anomalies and estimating fault severity. The hybridization of these methods offers enhanced diagnostic accuracy, combining the interpretability of physical models with the adaptability of ML.

Data-driven methods, such as auto-encoders and GANs, offer robust frameworks for detecting subtle anomalies in complex systems. These approaches, however, rely on high-quality labeled datasets, which are often limited. Conversely, physics-based methods, while highly interpretable, may struggle with scalability and complexity. Integrating these methods addresses their individual limitations, enabling comprehensive fault detection and prognosis.

DTs operationalize HIs, creating a continuous feedback loop between physical assets and their virtual counterparts. This integration facilitates predictive maintenance, reduces downtime, and extends system longevity. However, challenges persist, particularly in managing uncertainties arising from modeling assumptions, sensor inaccuracies, and operational variability. Employing uncertainty quantification techniques such as Bayesian methods and Monte Carlo simulations enhances the reliability of predictions, ensuring robustness across diverse operational conditions.

## 6. Conclusions

The integration of HIs and DTs signifies a major advancement in predictive maintenance and condition monitoring. By combining physics-based and data-driven approaches, DTs enhance the reliability of fault detection, diagnostics, and prognostics. HIs serve as essential tools for quantifying system health, enabling more accurate predictions of failure modes and RUL. However, challenges remain in improving fault classification and severity estimation and in managing uncertainties in real-world applications. Future research should focus on hybrid modeling, advanced AI-driven classification techniques, and enhanced uncertainty quantification in order to further optimize predictive maintenance strategies. As DT technology continues to evolve, its integration with robust HIs will play a pivotal role in the transition from traditional maintenance proceedings to performance-driven engineering.

## Data Availability

The data presented in this study are available on request from the corresponding author.
